# Notes and Notices

**Published:** 1896-10

**Authors:** 


### NOTES AND NOTICES.

THE STANDARD DICTIONARY.—Horatio Alger, Jr., New York City: "There can be but one opinion of the conduct of the competitor of the Standard Dictionary among fair-minded men. Had the editor omitted in the vocabulary the words objected to, it might and would have been declared incomplete. I attach great importance to the judgment of Chas. A. Dana in this matter. I make considerable use of the Standard Dictionary, and my estimate of its completeness and value grows from day to day." NOTE.—See THE HOMŒOPATHIC PHYSICIAN for January, 1896, page 53.—ED.

THE MAYWOOD BICYCLE.—Chicago, Ill., February 11th, 1896. I cheerfully recommend the "Maywood" Bicycle, as I consider it the best wheel on the market. I rode all last season, covering 7,000 miles of both boulevard and rough country riding, and the wheel is in as good condition as it was the day I bought it of you. You are at liberty to refer any one to me. W. E. HOPP, 358-366 Dearborn Street.

HERING ON TYPHOID was crowded out of our pages last month. We make up the omission this month with an increased number of pages.

